# Cerebellar motor and non-motor contributions to dystonia pathophysiology and treatment

**DOI:** 10.3389/dyst.2026.15082

**Published:** 2026-07-19

**Authors:** Linda H. Kim, Cheryl Brandenburg, Roy V. Sillitoe

**Affiliations:** 1 Department of Pathology and Immunology, Baylor College of Medicine, Houston, TX, United States,; 2 Cerebellum Science Center, Texas Children’s Hospital, Houston, TX, United States,; 3 Jan and Dan Duncan Neurological Research Institute, Of Texas Children’s Hospital, Houston, TX, United States,; 4 Department of Neuroscience, Baylor College of Medicine, Houston, TX, United States,; 5 Department of Pediatrics, Baylor College of Medicine, Houston, TX, United States,; 6 Development, Disease Models and Therapeutics Graduate Program, Baylor College of Medicine, Houston, TX, United States

**Keywords:** cerebellum, cognition, dystonia, movement, neuromodulation, sleep, neuroplasticity

## Abstract

Dystonia is a complex neurological disorder characterized by sustained or intermittent abnormal movements and/or postures. However, dystonia’s non-motor symptoms, particularly the sleep disturbances, are critical yet underexplored concerns that affect all ages. Here, we synthesize current knowledge on the motor and non-motor domains of dystonia, emphasizing the functional interconnections and plasticity within cerebellar motor, cognitive, and sleep-associated circuits to motivate more comprehensive and effective treatments that improve overall quality of life.

## Introduction

Dystonia is “a movement disorder characterized by sustained or intermittent abnormal movements, postures, or both [1].” However, this narrow diagnostic perspective does not account for the significance of non-motor symptoms that many individuals with dystonia experience [2, 3]. Sleep disturbances such as insomnia, fragmented sleep, and restless nights are common yet underexplored, despite their profound impact on quality of life [3, 4]. These unknowns highlight areas of opportunity to better understand the critical links between sleep, cognition, and motor function, underscoring the complex and interconnected nature of dystonia that extends its pathophysiological basis beyond being a purely motor condition.

Compounding dystonia’s complexity is the role of the cerebellum, which has been traditionally regarded as a motor structure having an important role in motor coordination and motor learning. However, emerging research has revealed cerebellar contributions to a broad range of non-motor functions, including sleep regulation, cognition, and sensory, emotional, and social processing [3, 5–9]. Conditions involving cerebellar dysfunction, including dystonia, Parkinson’s disease, autism spectrum disorder, and Cerebellar Cognitive Affective Syndrome (CCAS), among many others, exemplify the intricate interplay between cerebellar dysfunction and both motor and non-motor symptoms [9–13]. The overlapping challenges in these conditions suggest a broader, systems-level interaction between motor deficits and non-motor symptoms, with the cerebellum as a central hub. This mini review explores this complexity in the context of dystonia, highlighting a pressing need to better understand sleep, cognitive dysfunctions, and aberrant plasticity for the purpose of creating more comprehensive therapeutic strategies that will improve the overall outcomes for those affected.

## Motor symptoms in dystonia

Dystonic motor symptoms can occur in various forms. Focal dystonia affects isolated body parts, like the neck in cervical dystonia or the hand with writer’s cramp, to segmental dystonia affecting contiguous muscles, to generalized dystonia involving larger muscle groups distributed across the body [14, 15]. The severity of these symptoms often fluctuates, influenced by factors such as stress, fatigue, and voluntary movement [16]. The pathophysiology of these motor symptoms is complex and multifactorial, involving a broad “dystonia network” dysfunction rippling across the basal ganglia, thalamus, cortex, and cerebellum [17–20]. Although much of the literature has historically focused on task-specific focal dystonia, substantial evidence now demonstrates that network-level abnormalities also characterize non–task-specific focal dystonia and generalized dystonia, including widespread cortical dysfunction, impaired inhibition, and abnormal connectivity across basal ganglia-thalamo-cortical and cerebello-thalamo-cortical loops [17, 21, 22]. Reduced functional connectivity within the sensorimotor and inferior parietal cortices contributes to the polygenic risk of dystonia [23]. Functional and structural abnormalities across prefrontal–parietal, caudate, and thalamic regions likely reflect the underlying vulnerability to dystonia penetrance, whereas additional abnormalities in the premotorparietal-temporal and cerebellar regions are linked to dystonia manifestation with adaptive, compensatory and secondary changes [24]. Interestingly, both task-specific and non-task focal dystonia involve disruptions in the basal ganglia and cerebellum, yet their underlying mechanisms also diverge [25]. Compared to non-task dystonia, which involves more isolated dysfunction in the somatosensory cortex, task-specific focal dystonia engages cortical network dysfunction, with broader interactions among functional cortical domains whose involvement depends on the task [25]. For example, laryngeal dystonia is associated with the parietal cortex, inferior frontal gyrus, and anterior insula, while musician’s dystonia involves the primary and secondary sensorimotor cortex and middle frontal gyrus, reflecting the task specificity of different focal dystonias [26]. Importantly, dystonia is not restricted to primary motor circuits, and multimodal cortical regions that integrate motor, sensory, cognitive, and affective information are also implicated across dystonia subtypes, underscoring that dystonia reflects disturbances within a distributed network rather than isolated motor circuits.

## Non-motor symptoms in dystonia

Although the cerebellum has been traditionally appreciated for controlling smooth movement, its role has expanded profoundly in recent years to encompass supporting various non-motor behaviors, including cognition, memory, language, mood, and sleep [5]. Consequently, its dysfunction can be catastrophic across motor and non-motor domains in hereditary and acquired conditions. Non-motor symptoms are highly prevalent in dystonia [27–30]. Pain affects 55%–89% of individuals with cervical dystonia, and approximately 30%–40% across other focal forms [31]. Neuropsychiatric symptoms such as depression and anxiety are markedly elevated across idiopathic dystonia compared to controls, with cervical dystonia showing the highest burden, where up to 90% of individuals may meet criteria for a psychiatric diagnosis [32]. Accordingly, several links between dystonia and psychiatric disturbances have emerged. Psychiatric symptoms, particularly anxiety and depression, sometimes precede the onset of motor signs [33, 34] or appear in unaffected relatives [35], suggesting shared neurobiological substrates rather than secondary effects. Stress, self-consciousness, and physical factors like walking, carrying objects, or fatigue can exacerbate torticollis [16], blurring the boundary between motor and affective manifestations. The available evidence suggests that dystonia reflects network-level dysfunction rather than pathology confined to a single brain region, with abnormalities across the cortico-striato-thalamo-cortical and cortico-cerebello-thalamo-cortical circuits contributing to both motor and non-motor symptoms (see review [17]). However, our understanding of the pathophysiology of non-motor symptoms remains limited by the small sample sizes, heterogeneous methodologies, and overall paucity of dedicated studies. Future genetic, imaging, and longitudinal work will be essential to link motor and non-motor features and to identify the shared neural pathways that give rise to the full clinical phenotype (see key knowledge gaps: [30, 36]).

Sleep disturbances, including insomnia, restless sleep, and difficulty maintaining sleep [37–40], occur in roughly 40%–70% of individuals with adult-onset dystonia, particularly in cervical dystonia [37, 41]; and despite evidence that poor sleep can impair motor learning, cognitive function, and overall wellbeing [28, 42–45], they remain underappreciated (see reviews [3, 37, 41]). Research in individuals recovering from ischemic stroke suggests that sleep enhances implicit motor learning and motor memory consolidation, particularly when it occurs between practice and testing [46]. Thus, to improve motor outcomes in dystonia, it is important to consider how poor sleep affects cognition and how restoring sleep can support motor recovery. Understanding the interplay between motor and non-motor symptoms and how deficits in one functional domain can perpetuate dysfunction or be leveraged for recovery in others will be crucial for developing comprehensive therapies that address both the motor and non-motor symptoms in dystonia.

## Cerebellar contributions to dystonia

While the basal ganglia have traditionally been implicated in dystonia, growing evidence points to the cerebellum as a critical player, offering new insights into dystonia’s pathophysiology as a modulator of motor output and as a potential driver of the disease [9, 20, 47]. The cerebellum’s central role in motor control is well-established, with its dysfunction leading to ataxia, tremor, and dystonia, as we argue here as well [47–51]. Furthermore, mounting evidence supports the cerebellum’s influence over a wide range of cognitive, emotional, and sensory abnormalities [5]. Although motor and non-motor symptoms are often discussed separately, many cerebellar circuits that are a part of the dystonia network support both domains. Importantly, disruptions to cerebellar circuits can produce distinct symptom profiles depending on the timing, location, and nature of the dysfunction, such as developmental versus adult-onset, or genetic, acquired, or circuit-specific perturbations [5, 17, 52]. Nevertheless, irregular cerebellar spiking activity emerges as a consistent feature across different pharmacological, genetic, optogenetic, and circuit-based animal models of dystonia [47, 48, 51, 53–59]. This may involve aberrant spiking from Purkinje cells and/or the cerebellar nuclei, disrupted climbing fiber inputs, and altered cerebellar-thalamic communication [47, 53, 54, 59, 60]. Notably, cerebellar abnormalities in these models are not secondary to basal ganglia dysfunction but rather appear to play a causative role in symptom generation. For instance, in the DYT1 mouse model, irregular cerebellar output precedes dystonic movements and can be suppressed by pharmacological silencing of the cerebellar nuclei [58]. Indeed, cerebellar deep brain stimulation (DBS) can not only restore movement in mouse models of movement disorders such as dystonia, tremor, and ataxia [47, 49, 61], but it can also restore sleep deficits in dystonic mice [62]. This is particularly exciting since sleep disruptions across various mouse models of movement disorders have been shown to be dependent on cerebellar dysfunction but not motor symptoms [9, 12], and addressing only motor symptoms fails to rescue poor sleep in both mice [62] and humans [63]. In human patients with dystonia, dystonic movements resolve during sleep while sleep itself is disrupted [39]. This dissociation suggests that cerebellar dysfunction may independently drive sleep abnormalities in dystonia. Understanding how cerebellar networks regulate sleep while simultaneously suppressing dystonic motor activity may reveal new therapeutic opportunities. Together, these findings support the targeting of the cerebellum as a compelling dual-function therapeutic node for dystonia.

Recent work has also highlighted the cerebellum’s unique role in sleep-dependent memory consolidation and sensorimotor recalibration. During sleep, cerebellar-thalamocortical coherence changes dynamically across different sleep stages [64], but spike timing between the motor cortex (M1) and cerebellum remains temporally correlated across both active and offline states [65]. These findings suggest that neural patterns engaged during waking behavior may be replayed during sleep, potentially supporting offline learning and refinement of motor output. This idea is further supported by observations in neonatal rodents, where cerebellar output is required for the development of precise sensorimotor predictions during rapid eye movement (REM) sleep. Specifically, thalamic responses to spontaneous twitches, which are critical for sensorimotor development, become increasingly precise during postnatal development, and this process is disrupted by inactivation of the interposed nucleus [66]. While key motor and social neurodevelopmental milestones are affected by olivocerebellar silencing in an early-onset generalized dystonia mouse model [67], it remains unknown whether dystonic cerebellar circuits during development [68] fail to properly consolidate memories or whether sensorimotor predictions are negatively impacted. Directly examining sleep-dependent consolidation in early-onset dystonia model would help clarify whether restoring healthy sleep architecture, alongside guiding adaptive early motor patterns, could support more typical sensorimotor development.

## Systems-level neural network dysregulation in dystonia

At the neural systems level, a range of converging physiological abnormalities have been identified in dystonia, pointing to a widespread network dysfunction. These include abnormal basal ganglia firing patterns [69–72] and greater low-frequency oscillatory activity [73, 74], excessive low-frequency motor drive [75–78], disrupted sensorimotor processing [75, 79–83], reduced central inhibition [84], and increased synaptic plasticity [85, 86]. Together, these findings point to a failure in the coordinated activity within and between sensorimotor networks, leading to the emergence of involuntary, repetitive movements and abnormal postures. Importantly, the specific phenotype of a given dystonia likely reflects the site and nature of the underlying network disruption. For example, focal hand dystonia is often linked to abnormal cortical plasticity abnormalities and somatotopic disorganization, while generalized dystonia may involve deeper disturbances across basal ganglia-thalamo-cortical and cerebello-thalamo-cortical loops. Furthermore, the same clinical phenotypes can precipitate from diverse lesion sites, which tend to be functionally connected to common remote networks [87]. Across heterogeneous lesion sites, cervical dystonia consistently maps onto a cerebellar-somatosensory network that is also disrupted in idiopathic dystonia and preferentially connected to the most effective DBS targets [18]. Similarly, person-specific deviations in gray matter volume can be spread across distinct brain areas, yet they frequently converge on shared neural circuits or networks involved in specific brain functions [88]. Collectively, these findings help account for both the variability in clinical presentations and the common functional impairments observed, and they highlight how localized cerebellar disruptions may reverberate through distributed brain networks and influence widespread cellular functions and associated behaviors.

In this context, neuroanatomical data revealing extensive connections between the cerebellum and cerebral cortical association areas, such as the prefrontal cortex and limbic system, offer a compelling framework for understanding not only the motor but also the non-motor symptoms observed with cerebellar dysfunction [89–91]. It is likely that based on the complexity of cerebellar coordination, additional anatomical subdivisions, higher levels of multimodal input-output integration, and specialized encoding and plasticity mechanisms will be required. For instance, chemogenetic inhibition of Purkinje cells in lobule VI impairs coordinated activity across thalamic subregions, while inhibition in crus I disrupts coordination between sensorimotor and associative subnetworks during flexible, multiday behavior [92]. Furthermore, a single granule cell can receive mossy fiber inputs from multiple modalities, enabling integration across modalities [93–96]. Multimodal cortical influence may be further supported by cerebellar hubs within molecularly defined cerebellar modules of crus I, paraflocculus, and vermal regions IV/V and VI where mossy fiber terminals originating from the primary motor, sensory, and association cortical regions spatially overlap [97]. Although these multimodal hub regions are compelling candidates for multimodal integration relevant to dystonia, direct evidence of dysfunction within these specific cerebellar modules in dystonia models is not yet available. These findings highlight the importance of studying dystonia from a systems neuroscience perspective, one that leverages multi-site *in vivo* electrophysiological recordings, circuit-specific perturbations, and the mapping of aberrant network dynamics during behavior.

## Aberrant synaptic plasticity: a double-edged sword

Abnormal excessive synaptic plasticity may further contribute to dystonia pathology by failing to regulate plastic changes, and increasing maladaptive plasticity could worsen dystonic symptoms. Neurophysiological studies using techniques such as transcranial magnetic stimulation (TMS) have shown enhanced long-term potentiation-like responses, reduced intracortical inhibition, and impaired sensorimotor integration in patients with dystonia [85, 86, 98]. These abnormalities suggest a failure of homeostatic mechanisms that normally constrain plasticity to adaptive ranges. Further evidence comes from studies using paired associative stimulation (PAS), which reveal exaggerated and prolonged responses in dystonia patients compared to controls [99]. This heightened plasticity may underlie the persistence of maladaptive motor patterns and task-specificity seen in disorders like musician’s dystonia. In contrast, abnormal plasticity may not be essential for all forms of dystonia as patients with acquired hemidystonia show normal responses to PAS [100] and high-frequency repetitive sensory stimulation [101]. While aberrant plasticity might contribute to dystonia pathogenesis and progression, it could also provide an interesting opportunity for circuit recalibration. For example, pallidal DBS can normalize patterns of cortical excitability and restore more typical sensorimotor integration over time [102–104]. Emerging studies also point to the cerebellum as a key modulator of cortical excitability and plasticity. Cerebellar inhibition protocols, such as cerebellar TMS using continuous theta burst stimulation (cTBS) or inhibitory 1-Hz repetitive TMS over the cerebellum, have been shown to dampen overactive cortical plasticity and improve motor performance in patients with dystonia, though results have been mixed across subtypes ([105–109], see review [110]). A study targeting the cerebellum in writer’s cramp failed to produce a behavioral benefit, whereas similar protocols showed motor improvement in cervical dystonia [106, 111]. These discrepancies suggest that different dystonia subtypes may engage distinct cerebellar-cortical circuits and thus may respond differently to the same TMS protocols. Nevertheless, these approaches aim to recalibrate abnormal cerebello-thalamo-cortical communication and may offer a promising avenue to normalize impaired sensorimotor integration. While a broad range of stimulation parameters can be used, their effectiveness may depend also on the brain’s state, whether applied at rest or during task performance. Patients with dystonia show network abnormalities during both rest and active dystonic movement [25, 112–114], yet how these different brain states modulate the effectiveness of neuromodulatory interventions remains unclear. Recent findings from cerebellar DBS studies in post-stroke humans and a mouse model with dystonia, tremor, and ataxia suggest a synergy in motor recovery and unlocking of long-term benefits only when stimulation is paired with rehabilitative exercises [61, 115]. These findings reinforce the cerebellum’s potential to harness synaptic plasticity and maintain motor control fidelity in diseased states. Future research aimed at dissecting the temporal dynamics of circuit maturation, reorganization, and maladaptation will be critical for refining therapeutic strategies and for identifying windows of opportunity in which interventions may be most effective.

## Therapeutic recalibration–leveraging neuroplasticity for the treatment of dystonia

Despite having worse motor symptoms, pallidal DBS tends to be more effective in pediatric patients with dystonia than in adults. This raises important questions about how developmental plasticity and circuit maturation can shape symptom expression and treatment responsiveness. The disparity in outcomes may reflect a sensitive period during childhood in which heightened plasticity coincides with ongoing activity-dependent refinement of sensorimotor circuits. In addition, sleep disturbances can be less of a problem in some forms of early-onset dystonia [116], presenting a valuable model in which the integrity of sleep-dependent mechanisms that control motor learning and memory consolidation are potentially preserved. These insights underscore the importance of early diagnosis and intervention of early-life dystonic symptoms. Moreover, they argue for timing the therapeutic interventions to capitalize on different windows of plasticity to optimize outcomes.

**FIGURE 1:**
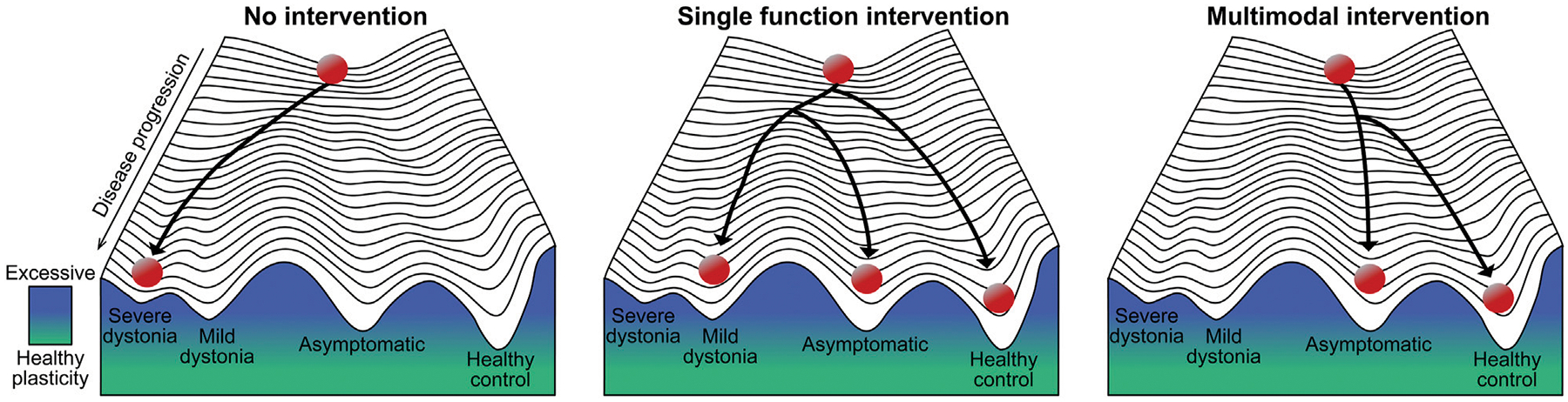
Therapeutic potential of different interventions for dystonia. Left: In the absence of intervention, dystonia progresses over time, leading to severe motor symptoms and abnormally heightened plasticity. Middle: A single-modality intervention, particularly if applied during a more amenable period, may delay, reduce, or even prevent the emergence of dystonic symptoms and associated pathophysiology. Right: Multimodal interventions may be more effective than single interventions, potentially harnessing excessive plasticity more effectively. This could result in a near-asymptomatic functional state or even restoration of functional networks approaching states seen in healthy individuals.

In genetic forms of dystonia, abnormal signaling may begin as early as embryogenesis, subtly altering the formation and function of key sensorimotor circuits. By contrast, acquired dystonia, particularly following perinatal insults, may interfere with circuits that were initially developing normally, only to be perturbed during a critical phase of refinement. Studies in neonatal mice have shown greater corticostriatal and corticothalamic coherences than thalamostriatal coherence during later postnatal ages, suggesting that cortical activity plays a dominant role in shaping early striatal function [117]. The cerebellum also plays a crucial role in shaping these developmental trajectories [118–121]. Cerebellar dysfunction, whether due to genetic mutation, developmental insult, or injury, can alter excitatory-inhibitory balance across the basal ganglia-thalamocortical network, seeding a cascade of maladaptive plasticity. This destabilization can reinforce abnormal movement patterns over time through aberrant synaptic strengthening and pruning [98]. In conditions like cerebral palsy and acquired hemidystonia, symptoms often emerge only after a delay from the initial cerebellar injury, suggesting a time-dependent unfolding of network-wide reorganization and maladaptive processes [122, 123]. In a mouse model of dystonia created by lesioning the cerebellar output pathways, DBS yielded robust immediate therapeutic effects when applied to the centrolateral thalamus, a first-order downstream target of cerebellar projections. However, targeting the dorsal striatum, a second-order node in the same circuit, failed to produce similar benefits [124]. Phenotype-specific differences in optimal DBS stimulation networks have been demonstrated in human patients with dystonia, where modulation of the striatopallidofugal axis appears most effective for cervical dystonia, whereas modulation of pallidothalamic bundles better predicts improvement in generalized dystonia, with both subtypes converging on a shared multisynaptic network involving the cerebellum and somatomotor cortex [125]. These results underscore the importance of precise circuit-level targeting in optimizing DBS outcomes. Identifying pathways through which cerebellar disturbances propagate, and understanding when these circuits are amenable to modulation, will be critical for designing targeted neuromodulation strategies aimed at restoring balance in the circuit before pathological plasticity becomes entrenched (Figure 1). Importantly, harnessing neuroplasticity remains a promising therapeutic strategy even in adulthood, as the cerebellum plays a critical role in motor learning and adaptation to changing environments and retains a high capacity for plasticity even following injury [115, 126–128].

Taken together, these findings position the cerebellum not only as a key driver of dystonia-related pathology but also as a promising target for therapeutic recalibration. Future neuromodulation strategies might benefit from leveraging sleep-dependent plasticity, developmentally informed intervention timing, and circuit-specific stimulation paradigms to optimize outcomes. By harnessing both spontaneous and induced forms of neuroplasticity, we may begin to think of ways to reshape abnormal motor networks (structure and function) and offer a fresh perspective for effective, personalized treatment across the lifespan.

## Discussion

### Therapeutic implications and future research directions

Dystonia is a multifaceted disorder that extends beyond its motor symptoms to include significant non-motor challenges. The cerebellum emerges as a critical player in both domains, influencing sensorimotor integration, sleep regulation, and cognitive processing. Moreover, linking cerebellar dysfunction to therapeutic strategies offers an opportunity to develop innovative treatments not only for dystonia but also for a broader range of neurological and psychiatric conditions [129]. Advancements in our understanding of biomarkers and electrophysiological signatures [19, 130] are paving the way for early diagnosis and more targeted treatments of dystonia. Nevertheless, the establishment of a definitive biomarker for dystonia still remains an active area of investigation. Theta oscillations in the globus pallidus have been shown to be strongly associated with dystonic symptoms [73, 75, 77, 82], and the elevated theta interactions with weakened oscillations in the alpha, low beta, and gamma bands dynamically contribute to high contraction dystonic symptoms [131]. Restoring the abnormal balance between these oscillations could alleviate dystonic symptoms by rebalancing the flow of motor information in the basal ganglia network [132–135]. Interestingly, low beta/high beta power ratio positively correlated with both high and low muscle contractions during dystonic episodes, suggestive of it being a good biomarker for dystonia [131], but this remains to be experimentally tested. In addition, prominent alpha oscillations in the dentate nucleus in three patients with acquired dystonia have been reported, but the significance of these observations requires further investigation [136]. Together, these oscillatory signatures raise the possibility that specific frequency-band abnormalities could serve as biomarkers for symptom severity or real-time control signals for adaptive DBS, although their predictive value for prognosis, treatment response, or target selection has not yet been determined. At present, these biomarkers are best viewed as promising candidates for guiding closed-loop neuromodulation rather than established tools for diagnosis or therapeutic stratification. Nevertheless, these electrophysiological irregularities highlight the complex and heterogeneous nature of dystonia pathophysiology, reinforcing the need for a more personalized approach to DBS therapy, one that accounts for differences in pathological functional networks [125, 137] as well as variations in pallidal firing rates and patterns across dystonia etiologies and phenotypes [72]. Moreover, sleep quality, often overlooked, holds immense potential as an early indicator of circuit dysfunction and a synergist of recovery. Future research should also focus on elucidating the precise mechanisms linking cerebellar dysfunction to the motor and non-motor aspects of dystonia over time. By addressing both motor and non-motor symptoms, we can move closer to improving the overall quality of life for individuals with dystonia.
